# Pre-Illness Isoflavone Consumption and Disease Risk of Ulcerative Colitis: A Multicenter Case-Control Study in Japan

**DOI:** 10.1371/journal.pone.0110270

**Published:** 2014-10-14

**Authors:** Satoko Ohfuji, Wakaba Fukushima, Kenji Watanabe, Satoshi Sasaki, Hirokazu Yamagami, Masakazu Nagahori, Mamoru Watanabe, Yoshio Hirota, for the Japanese Case-Control Study Group for Ulcerative Colitis

**Affiliations:** 1 Department of Public Health, Osaka City University Faculty of Medicine, Osaka, Japan; 2 Department of Gastroenterology, Osaka City University Faculty of Medicine, Osaka, Japan; 3 Department of Social and Preventive Epidemiology, School of Public Health, The University of Tokyo, Tokyo, Japan; 4 Department of Gastroenterology and Hepatology, Tokyo Medical and Dental University, Tokyo, Japan; 5 Clinical Epidemiology Research Center, Medical Co. LTA, Fukuoka, Japan; University Hospital Llandough, United Kingdom

## Abstract

**Introduction:**

Previous studies have suggested that estrogens play a role in the development of ulcerative colitis (UC). Because isoflavones have a similar structure to 17β-estradiol, dietary consumption of isoflavones may have similar influences on the development of UC. We examined the association between pre-illness isoflavone consumption and the risk of UC.

**Materials and Methods:**

We conducted a hospital-based case control study, and compared the dietary habits of 126 newly diagnosed UC cases with those of 170 age- and gender-matched hospital controls. Information on dietary factors was collected using a self-administered diet history questionnaire. To consider potential changes in dietary habits due to disease symptoms, the habits were assessed separately during the previous 1 month and at 1 year before the recruitment.

**Results:**

In the assessment of dietary habits during the previous 1 month, the highest tertile of isoflavone consumption revealed an increased odds ratio (OR) for UC (OR = 2.79; 95% confidence interval (CI), 1.39–5.59; Trend P = 0.004). A significant association was also observed for the dietary assessment at 1 year before, when most UC cases had not yet experienced their first disease symptoms (OR = 2.06; 95% CI, 1.05–4.04; Trend P = 0.04). Associations were more pronounced in females (OR in highest tertile of isoflavone consumption at 1 year before = 4.76; 95% CI, 1.30–17.5; Trend P = 0.02) but were obscured in males (corresponding OR = 1.21; 95% CI, 0.49–3.01; Trend P = 0.63).

**Conclusions:**

Dietary isoflavone consumption may be associated with an increased risk of UC, particularly in females. Prospective cohort studies are warranted to confirm these findings.

## Introduction

In Japan, the prevalence of ulcerative colitis (UC) has been increasing over the last two decades [1], [2] and the age-standardized prevalence of UC was 63.6 per 100,000 persons in 2005 [3]. However, the etiology and pathogenesis of UC have remained largely unclear. Although several studies have confirmed an important role of genetic predisposition in UC [4], the number of UC patients with a family history of inflammatory bowel disease remains low [5], and the rising incidence against a background of stable prevalence of the genetic predisposition [5], [6] has suggested the importance of environmental factors in the disease etiology.

Several potential environmental or external risk factors have been reported to date, including oral contraceptive use [7]–[9]. Two meta-analyses have provided evidence for a modest association between the use of oral contraceptive agents and the development of UC [10], [11]. Another study indicated that hormone-replacement therapy increased the risk of UC among postmenopausal women [12]. Such findings suggest that pathways related to estrogens might mediate the pathogenesis of UC.

Isoflavones have a similar structure to 17β-estradiol. Daidzein and genistein are the main isoflavones present in soybeans, and possess the ability to bind to estrogen receptors [13], [14]. Dietary consumption of isoflavones has therefore been suggested to potentially exert a similar influence to estrogens on the development of UC. In addition, extensive animal studies showing the effect of isoflavones on immune parameters have suggested the feasibility of genistein and daidzein exerting immunological effects in humans [15]. However, these possible associations have yet to be evaluated.

We therefore sought to examine the association between isoflavone consumption and risk of UC development, using a multicenter case-control study in Japan. Since UC patients are likely to change dietary habits following the onset of disease symptoms, pre-illness dietary habits would be important to examine in considering issues of causality. Thus, the present hospital-based case-control study enrolled newly diagnosed UC patients as cases, and dietary habits both during the previous 1 month and at 1 year before recruitment were assessed separately as candidate pre-illness dietary habits.

## Materials and Methods

### Selection of Cases and Controls

Between September 2008 and March 2014, a multicenter case-control study was conducted in Japan, to investigate risk factors for the development of UC. Newly diagnosed cases of UC were recruited at 38 collaborating hospitals, in which the gastroenterologists were members of the Research Committee of Inflammatory Bowel Disease. Eligible cases were patients who were newly diagnosed with UC in those hospitals and whose age at diagnosis was less than 80 years. In the case of UC patients referred from neighboring associated hospitals, patients who had been diagnosed within the preceding 3 months were regarded as eligible. Collaborating gastroenterologists were responsible for UC diagnosis, in accordance with the following diagnostic criteria proposed by the Research Committee of Inflammatory Bowel Disease in 2008: patients should have symptoms (or episodes) of persistent/repetitive bloody diarrhea or mucous bloody stool and also characteristic findings of the disease on endoscopy, barium enema study, or histological study; infectious, radiation-induced, ischemic, or granulomatous colitis should be excluded [16]. These patients were asked to participate in the present study as soon as possible after diagnosis.

Two matched controls for each case with UC were sought in the same hospital as the enrolled case of UC. We encouraged collaborating hospitals to select two controls: one from the department of digestive diseases, and the other from another department (i.e., orthopedic surgery, internal medicine, ophthalmology, dermatology, otolaryngology, etc.). Conditions for matching were gender and age (within the same 5-year age group, as follows: 10–14 years, 15–19 years, 20–24 years, 25–29 years, and so on). Exclusion criteria were follows: presence of malignant neoplasm; lasting symptoms of diarrhea and/or abdominal pain for more than 1 week; or history of inflammatory bowel disease. Each collaborating hospital was asked to provide two sets of these cases and controls every year.

The study protocol was approved by the ethics committees at Osaka City University Faculty of Medicine and Tokyo Medical and Dental University, and was performed in accordance with the Declaration of Helsinki. Written informed consent was obtained from all subjects prior to participation. In cases where the subject was less than 20 years old, written informed consent was obtained from the subject's legal representative.

### Information Collection

The following clinical findings of UC patients were reported by the gastroenterologists-in-charge using a standardized questionnaire: date at symptom onsets; date at first visit to the hospital; disease severity at diagnosis (mild, moderate, severe, or fulminant); location of disease at diagnosis (rectum, colon, cecum, or ileum); and parenteral complications. Afterwards, age at symptom onset, duration from symptom onset to recruitment, and duration from first visit to the hospital to recruitment were calculated from the date of birth, date at symptom onset, date at first visit to the hospital and recruitment date. Disease severity, based on the criteria proposed by the Research Committee of Inflammatory Bowel Disease, was regarded as “mild” when the frequency of defecation was 4 times/day or less, bloody diarrhea or mucous bloody stool was absent or minimal, and systemic symptoms were absent, as “severe” when the frequency of defecation was 6 times/day or more, severe bloody diarrhea or mucous bloody stool was present, and systemic symptoms (fever, tachycardia, anemia, etc.) were apparent, and as “moderate” when the features were intermediate between “mild” and “severe”. In particular, patients with “severe” disease and those showing extremely severe symptoms (bloody stools of about 15 times/day or more, persistent high fever of 38°C or higher, increase in leukocyte count to 10,000/mm^3^ or more, and severe abdominal pain) were classified as having “fulminant” disease [16].

In addition, study subjects were asked to fill out a set of 2 self-administered, mail-back questionnaires. One questionnaire was used to obtain information about demographic factors, past medical history including appendicitis, family history of UC, smoking (never, ever, or current), alcohol drinking (never, ever, or current), and, for females, menopausal status and use of exogenous female hormones including oral contraceptives and hormone-replacement therapy.

The other questionnaire was a validated self-administered diet-history questionnaire (DHQ), which assessed dietary habits during the previous 1 month. In this instrument, estimates of daily consumption for a total 150 food items, energy, and selected nutrients were calculated using an ad hoc computer algorithm for the DHQ. Detailed descriptions of the methods used for calculating dietary consumption and the validity of the DHQ have been published elsewhere [17]–[20]. In this questionnaire, the frequencies of intake for 6 soy products (tofu, tofu products such as deep-fried tofu and fried bean curd, fermented soybeans, boiled soybeans, miso, and miso soup) were asked, and daily consumptions for each food item were estimated. Total consumption of soy products was considered as the sum of these 6 food items. Isoflavone consumption from these soy products, which included daidzein and genistein, was estimated according to previously published studies [21], [22]. In the present study, the sum of daidzein and genistein consumptions was regarded as the isoflavone consumption. If study subjects answered that they had changed their dietary habits within 1 year, we asked for further information about their dietary habits at 1 year before recruitment using the same questionnaire. Previous studies have demonstrated that retrospective recall of dietary intake for the distant past using a self-administered food frequency questionnaire yielded moderate correlation coefficients with the original reports at that time, in terms of both dietary and nutrient consumptions [23], [24].

### Statistical Analysis

Energy-adjusted intake by the density method was used for the analyses. The chi-square test, Wilcoxon rank-sum test and Student's t-test were used to compare characteristics between cases and controls. Intakes of selected foods and nutrients were categorized into tertiles according to the distribution of control subjects. Logistic regression model was used to calculate odds ratios (ORs) and 95% confidence intervals (95% CIs) for UC development. Trends for association were assessed by assigning ordinal scores to a single dietary variable. Variables showing p-values less than 0.1 or that seemed likely to correlate with isoflavone consumption were considered as potential confounders for adjustment.

In addition, to examine gender-specific associations between isoflavone consumption and UC development, stratified analyses by gender were also conducted. When we assessed associations in females, menopausal status and use of exogenous female hormones were also considered as potential confounders.

All tests were two-sided. All analyses were performed using SAS version 9.3 software (SAS Institute, Cary, NC, USA).

## Results

Among the 150 UC cases and 206 controls enrolled, 126 cases and 170 controls responded to the questionnaire (response rates, 84% for cases and 83% for controls). The voluntary participation of study subjects thus resulted in some variations in matched status. Although 44 sets (44 cases and 88 controls) maintained the initial matching condition, 38 cases had only one matched control each, and 44 cases and 44 controls had no corresponding counterparts for pairing. Thus, to increase the statistical power, further main analyses were conducted in all 126 cases and 170 controls who responded to the questionnaire, using unconditional logistic regression model with adjustment for matching factors (age categories and gender).

Table 1 shows the clinical characteristics of the newly diagnosed UC cases. Mean age at recruitment was 41.2 years. About 90% of cases had experienced the first symptoms of disease within the preceding 11 months. About 40% suffered from mild disease, whereas 18% had severe disease.

**Table 1:** Clinical characteristics in patients with newly diagnosed ulcerative colitis (N = 126).*

Characteristics		n (%)
Age (years)	Mean (SD)	41.2 (14.6)
	<30	30 (24)
	30–39	33 (26)
	40–49	31 (25)
	50+	32 (25)
Age at symptom onsets (years)	Mean (SD)	41.2 (14.8)
	<30	24 (27)
	30–39	20 (22)
	40–49	23 (26)
	50+	23 (26)
	Unknown	36
Duration from symptom onsets (months)	Median (range)	2.4 (0–276)
	<4	59 (66)
	4–11	21 (23)
	12+	10 (11)
	Unknown	36
Duration from first visit to the hospital (months)	Median (range)	1.2 (0–651.6)
	<4	104 (85)
	4–11	14 (11)
	12+	4 (3)
	Unknown	4
Disease severity	Mild	35 (40)
	Moderate	37 (42)
	Severe	16 (18)
	Fulminant	0 (0)
	Unknown	38
Location of disease	Rectum	19 (22)
	Colon	39 (44)
	Cecum	27 (31)
	Ileum	3 (3)
	Unknown	38
Parenteral complications	Present	1 (1)
	Unknown	40

SD, standard deviation.

^*^Data expressed as n (%) unless otherwise indicated.

As for controls, we confirmed that they were derived from the departments of digestive diseases and other departments, at a ratio of approximately 1 to 1. The most frequent digestive disease was liver diseases (n = 36), followed by upper digestive diseases (n = 24) and colon diseases (n = 19). The most frequent disease from other departments was orthopedic disease (n = 26), followed by ophthalmologic disease (n = 7), chronic renal disease (n = 7), and others (n = 51).

Table 2 shows the background characteristics of study subjects. Age and gender distributions were similar between cases and controls. However, cases had a lower body mass index, less frequent history of appendicitis, but more frequent family history of UC than controls. In addition, significant differences were identified in smoking and drinking habits. Regarding dietary intake, cases consumed a higher total amount of soy products than controls, for assessments of both the previous 1 month and at 1 year before recruitment. In addition, isoflavone consumption including daidzein and genistein was also higher in cases both during the previous 1 month and at 1 year before.

**Table 2:** Characteristics of the 126 cases and 170 controls.

Variables		Cases	Controls	P †
		n (%)*	n (%)*	
Age (years)	<30	30 (24)	36 (21)	0.39
	30–39	33 (26)	41 (24)	
	40–49	31 (25)	43 (25)	
	50+	32 (25)	50 (29)	
Gender	Male	73 (58)	89 (52)	0.34
	Female	53 (42)	81 (48)	
Body mass index (kg/m^2^)	<21.0	67 (53)	58 (34)	0.001
	21.0–23.6	32 (25)	55 (32)	
	23.7+	27 (21)	57 (34)	
History of appendicitis	Present	8 (6)	29 (17)	0.006
Family history of ulcerative colitis	Present	9 (7)	5 (3)	0.09
Smoking habit	Never	63 (50)	98 (58)	0.003
	Ever	45 (36)	32 (19)	
	Current	18 (14)	40 (24)	
Drinking habit	Never	34 (27)	61 (36)	0.01
	Ever	25 (20)	14 (8)	
	Current	67 (53)	95 (56)	
Age at menarche (years)	Mean (SD)	12.7 (1.4)	12.7 (1.6)	0.76
Postmenopausal status	Present	13/53 (25)	26/81 (32)	0.35
Age at menopause (years)	Mean (SD)	50.2 (5.8)	48.3 (6.3)	0.24
	Unknown	0	2	
Use of exogenous female hormones	Ever	8/52 (15)	17/79 (22)	0.38
	Unknown	1	2	
*Dietary intake during the previous 1 month* §				
Total energy (kJ)	Mean (SD)	8590 (2761)	8541 (3314)	0.43
Total soy product (g/4184 kJ)	Mean (SD)	24.7 (17.8)	19.3 (14.8)	0.001
Tofu (g/4184 kJ)	Mean (SD)	13.7 (10.9)	9.9 (9.5)	0.001
Tofu product (g/4184 kJ)	Mean (SD)	0.9 (1.9)	1.0 (1.9)	0.15
Fermented soybeans (g/4184 kJ)	Mean (SD)	4.3 (6.2)	3.8 (6.2)	0.28
Boiled soybeans (g/4184 kJ)	Mean (SD)	1.7 (2.4)	1.7 (2.8)	0.66
Miso (g/4184 kJ)	Mean (SD)	1.3 (7.7)	0.4 (0.9)	0.71
Miso soup (g/4184 kJ)	Mean (SD)	2.9 (3.2)	2.5 (2.6)	0.40
Isoflavone (mg/4184 kJ)	Mean (SD)	13.6 (10.1)	11.1 (9.4)	0.005
Daidzein (mg/4184 kJ)	Mean (SD)	5.2 (3.8)	4.2 (3.5)	0.005
Genistein (mg/4184 kJ)	Mean (SD)	8.5 (6.3)	6.9 (5.8)	0.005
*Dietary intake at 1 year before* §				
Total energy (kJ)	Mean (SD)	8859 (2837)	8624 (3451)	0.12
Total soy product (g/4184 kJ)	Mean (SD)	22.8 (17.6)	19.3 (14.7)	0.04
Tofu (g/4184 kJ)	Mean (SD)	11.8 (10.4)	10.2 (9.9)	0.11
Tofu product (g/4184 kJ)	Mean (SD)	0.8 (1.5)	1.1 (1.9)	0.20
Fermented soybeans (g/4184 kJ)	Mean (SD)	4.4 (6.4)	3.5 (6.1)	0.13
Boiled soybeans (g/4184 kJ)	Mean (SD)	1.8 (2.6)	1.6 (2.6)	0.20
Miso (g/4184 kJ)	Mean (SD)	1.3 (7.7)	0.4 (0.9)	0.71
Miso soup (g/41841kJ)	Mean (SD)	2.7 (2.6)	2.5 (2.6)	0.36
Isoflavone (mg/4184 kJ)	Mean (SD)	13.0 (10.1)	10.9 (9.1)	0.02
Daidzein (mg/4184 kJ)	Mean (SD)	4.9 (3.8)	4.1 (3.4)	0.02
Genistein (mg/4184 kJ)	Mean (SD)	8.1 (6.3)	6.7 (5.6)	0.02

SD, standard deviation.

^*^Data expressed as n (%) unless otherwise indicated.

† The χ^2^ test or Wilcoxon rank-sum test were employed where appropriate.

§ Nutrient intake was adjusted for total energy intake using the density method.

After adjustment for potential confounders, consumption of total soy products during the previous 1 month revealed a significantly increased OR in the highest tertile (Table 3). The association was significantly dose-respondent (Trend P = 0.007). Among individual soy products, only tofu consumption showed a significantly higher OR in the highest tertile (OR = 1.98; 95%CI, 1.06–3.70). As for nutrients, higher isoflavone consumption (both daidzein and genistein) was associated with increased ORs with a trend towards (OR in highest tertile = 2.79; 95%CI, 1.39–5.59; Trend P = 0.004). Associations with isoflavone remained significant even for the assessment of consumption at 1 year before recruitment, although the OR in the highest tertile of isoflavone consumption was somewhat lower than that during the previous 1 month (OR = 2.06; 95%CI, 1.05–4.04; Trend P = 0.04). To exclude the possibility of reverse causality even in the assessment of dietary habits at 1 year before, sensitivity analyses were conducted, with analyzed UC cases limited to those patients who experienced the first symptom within the preceding 11 months (80 cases, 170 controls). However, the results were almost unchanged (OR in highest tertile of isoflavone consumption at 1 year before = 2.66; 95%CI, 1.18–6.03; Trend P = 0.021). When we examined the association between isoflavone consumption and localization of UC, the association was more clearly observed for disease reaching the cecum or ileum (OR in highest tertile of isoflavone consumption at 1 year before = 4.60; 95%CI, 1.18–18.0; Trend P = 0.035), but was attenuated for disease located in the rectum only (OR in highest tertile of isoflavone consumption at 1 year before = 2.69; 95%CI, 0.73–9.93; Trend P = 0.110).

**Table 3:** Odds ratios of soy product intake and isoflavone intake for development of ulcerative colitis.

Variables	During the previous 1 month				1 year before			
	Tertile			P for trend	Tertile			P for trend
	1 (lowest)	2	3 (highest)		1 (lowest)	2	3 (highest)	
**Total soy product**								
Daily intake (g/4184 kJ)*	<11.3	11.3–21.8	21.9+		<11.1	11.1–22.2	22.3+	
No. cases/controls	27/57	37/56	62/57		30/57	45/56	51/57	
Crude OR (95%CI)	1.00	1.40 (0.75–2.59)	2.30 (1.28–4.11)	0.004	1.00	1.53 (0.85–2.76)	1.70 (0.95–3.04)	0.08
Multivariate OR (95%CI)†	1.00	1.32 (0.67–2.60)	2.45 (1.25–4.79)	0.007	1.00	1.51 (0.79–2.89)	1.80 (0.92–3.53)	0.09
**Tofu**								
Daily intake (g/4184 kJ)*	<4.68	4.68–10.85	10.86+		<4.6	4.6–11.4	11.5+	
No. cases/controls	31/57	32/56	63/57		35/57	43/56	48/57	
Crude OR (95%CI)	1.00	1.05 (0.57–1.95)	2.03 (1.16–3.58)	0.01	1.00	1.25 (0.70–2.23)	1.37 (0.78–2.42)	0.28
Multivariate OR (95%CI)†	1.00	0.89 (0.45–1.75)	1.98 (1.06–3.70)	0.02	1.00	1.12 (0.59–2.11)	1.29 (0.69–2.43)	0.43
**Tofu product**								
Daily intake (g/4184 kJ)*	0	0.001–1.66	1.67+		0	0.01–1.62	1.63+	
No. cases/controls	84/94	18/38	24/38		80/91	20/39	26/40	
Crude OR (95%CI)	1.00	0.53 (0.28–0.99)	0.71 (0.39–1.28)	0.12	1.00	0.58 (0.32–1.08)	0.74 (0.42–1.32)	0.18
Multivariate OR (95%CI)†	1.00	0.59 (0.30–1.17)	0.66 (0.34–1.27)	0.13	1.00	0.70 (0.36–1.37)	0.69 (0.36–1.32)	0.20
**Fermented soybeans**								
Daily intake (g/4184 kJ)*	0	0.01–3.47	3.48+		0	0.01–3.12	3.13+	
No. cases/controls	39/67	46/51	41/52		36/67	47/51	43/52	
Crude OR (95%CI)	1.00	1.55 (0.88–2.72)	1.36 (0.77–2.39)	0.28	1.00	1.72 (0.97–3.02)	1.54 (0.87–2.73)	0.14
Multivariate OR (95%CI)†	1.00	1.53 (0.83–2.83)	1.41 (0.75–2.67)	0.27	1.00	1.65 (0.89–3.06)	1.61 (0.85–3.06)	0.13
**Boiled soybeans**								
Daily intake (g/4184 kJ)*	0	0.01–1.72	1.73+		0	0.01–1.604	1.605+	
No. cases/controls	48/67	35/51	43/52		40/64	38/53	48/53	
Crude OR (95%CI)	1.00	0.96 (0.54–1.69)	1.15 (0.67–2.00)	0.62	1.00	1.15 (0.65–2.04)	1.45 (0.83–2.53)	0.19
Multivariate OR (95%CI)†	1.00	0.93 (0.49–1.75)	1.12 (0.60–2.09)	0.74	1.00	1.12 (0.59–2.10)	1.51 (0.80–2.83)	0.21
**Miso**								
Daily intake (g/4184 kJ)*	0	0.01–1.374	1.375+		0	0.01–1.374	1.375+	
No. cases/controls	97/134	15/18	14/18		97/134	15/18	14/18	
Crude OR (95%CI)	1.00	1.15 (0.55–2.40)	1.07 (0.51–2.27)	0.76	1.00	1.15 (0.55–2.40)	1.07 (0.51–2.27)	0.76
Multivariate OR (95%CI)†	1.00	1.62 (0.73–3.60)	1.28 (0.54–3.02)	0.35	1.00	1.62 (0.73–3.60)	1.28 (0.54–3.02)	0.35
**Miso soup**								
Daily intake (g/4184 kJ)*	<1.009	1.009–2.97	2.98+		<0.98	0.98–2.80	2.81+	
No. cases/controls	33/57	52/56	41/57		31/57	51/56	44/57	
Crude OR (95%CI)	1.00	1.60 (0.91–2.84)	1.24 (0.69–2.24)	0.50	1.00	1.68 (0.94–2.99)	1.42 (0.79–2.56)	0.27
Multivariate OR (95%CI)†	1.00	1.42 (0.76–2.66)	1.23 (0.64–2.38)	0.54	1.00	1.33 (0.70–2.50)	1.47 (0.76–2.84)	0.25
**Isoflavone**								
Daily intake (mg/4184 kJ)*	<6.0	6.0–12.2	12.3+		<6.01	6.01–12.06	12.07+	
No. cases/controls	25/57	42/56	59/57		28/57	45/56	53/57	
Crude OR (95%CI)	1.00	1.71 (0.92–3.17)	2.36 (1.30–4.28)	0.005	1.00	1.64 (0.90–2.98)	1.89 (1.05–3.40)	0.04
Multivariate OR (95%CI)†	1.00	1.78 (0.91–3.50)	2.79 (1.39–5.59)	0.004	1.00	1.75 (0.91–3.38)	2.06 (1.05–4.04)	0.04
**Daidzein**								
Daily intake (mg/4184 kJ)*	<2.28	2.28–4.66	4.67+		<2.26	2.26–4.5	4.6+	
No. cases/controls	25/57	42/56	59/57		28/57	45/56	53/57	
Crude OR (95%CI)	1.00	1.71 (0.92–3.17)	2.36 (1.30–4.28)	0.005	1.00	1.64 (0.90–2.98)	1.89 (1.05–3.40)	0.04
Multivariate OR (95%CI)†	1.00	1.74 (0.89–3.42)	2.73 (1.37–5.46)	0.005	1.00	1.73 (0.90–3.36)	2.04 (1.04–4.01)	0.04
**Genistein**								
Daily intake (mg/4184 kJ)*	<3.7	3.7–7.6	7.7+		<3.7	3.7–7.45	7.46+	
No. cases/controls	25/57	43/56	58/57		28/57	45/56	53/57	
Crude OR (95%CI)	1.00	1.75 (0.95–3.24)	2.32 (1.28–4.21)	0.006	1.00	1.64 (0.90–2.98)	1.89 (1.05–3.40)	0.04
Multivariate OR (95%CI)†	1.00	1.83 (0.94–3.59)	2.72 (1.36–5.46)	0.005	1.00	1.75 (0.91–3.37)	2.06 (1.05–4.07)	0.04

OR, odds ratio; CI, confidence interval.

^*^Tertiles were based on intake in g/4184 kJ or mg/4184 kJ adjusted for energy intake using the density method.

† Adjusted for age, gender, body mass index, history of appendicitis, family history of ulcerative colitis, smoking and alcohol drinking status.

Table 4 shows the results of gender-stratified analyses regarding the association between isoflavone consumption and UC development. Increased ORs in highest tertiles were more clearly observed in females. In particular, an association with isoflavone consumption at 1 year before was obviously increased in females (OR in highest tertile = 4.76; 95%CI, 1.30–17.5; Trend P = 0.02) but was obscured in males (the corresponding OR = 1.21; 95%CI, 0.49–3.01; Trend P = 0.63). Increased ORs in females were unchanged even after adjusting for menopausal status and use of exogenous female hormones.

**Table 4:** Odds ratios of isoflavone intake for development of ulcerative colitis, stratified by gender.

Variables	During the previous 1 month				1 year before			
	Tertile			P for trend	Tertile			P for trend
	1 (lowest)	2	3 (highest)		1 (lowest)	2	3 (highest)	
**Isoflavone**								
OR (95%CI) in male*	1.00	1.50 (0.66–3.38)	2.15 (0.86–5.38)	0.099	1.00	1.27 (0.57–2.87)	1.21 (0.49–3.01)	0.63
OR (95%CI) in female*	1.00	2.44 (0.61–9.67)	4.43 (1.21–16.3)	0.02	1.00	3.29 (0.85–12.7)	4.76 (1.30–17.5)	0.02
OR (95%CI) in female†	1.00	2.53 (0.64–10.1)	4.61 (1.26–16.9)	0.02	1.00	3.06 (0.79–11.8)	4.44 (1.20–16.4)	0.03
**Daidzein**								
OR (95%CI) in male*	1.00	1.54 (0.68–3.47)	2.05 (0.83–5.08)	0.11	1.00	1.17 (0.51–2.65)	1.16 (0.47–2.90)	0.72
OR (95%CI) in female*	1.00	2.19 (0.56–8.65)	4.08 (1.14–14.6)	0.02	1.00	3.64 (0.95–14.0)	5.03 (1.38–18.3)	0.02
OR (95%CI) in female†	1.00	2.25 (0.57–8.93)	4.21 (1.18–15.1)	0.02	1.00	3.41 (0.89–13.1)	4.71 (1.29–17.2)	0.02
**Genistein**								
OR (95%CI) in male*	1.00	1.57 (0.70–3.53)	2.01 (0.80–5.09)	0.13	1.00	1.23 (0.55–2.77)	1.27 (0.51–3.20)	0.58
OR (95%CI) in female*	1.00	2.44 (0.61–9.67)	4.43 (1.21–16.3)	0.02	1.00	3.48 (0.90–13.4)	4.56 (1.25–16.7)	0.03
OR (95%CI) in female†	1.00	2.53 (0.64–10.1)	4.61 (1.26–16.9)	0.02	1.00	3.25 (0.84–12.6)	4.24 (1.15–15.6)	0.04

OR, odds ratio; CI, confidence interval.

^*^Adjusted for age, body mass index, history of appendicitis, family history of ulcerative colitis, smoking and alcohol drinking status.

† Further adjusted for menopausal status and use of exogenous female hormones.

In addition, conditional logistic regression models were employed in which analyzed subjects were limited for the matched sets (82 cases and 126 controls). As a result, the increased ORs of isoflavone consumption at 1 year before were similarly observed, although lower statistical power brought about the broader confidence intervals (OR in highest tertile = 1.94; 95%CI, 0.76–4.98; Trend P = 0.16). In the gender-stratified analyses, we could not obtain meaningful ORs in females, since only 1 case fell into the lowest tertile of isoflavone consumption. When lowest and intermediate tertiles were combined to regard as a reference category, ORs in highest tertile of isoflavone consumption at 1 year before were 0.66 (95%CI, 0.19-2.35) in males and 4.90 (95%CI, 1.18–20.3) in females, respectively.

## Discussion

The results of the present case-control study showed a possible association between higher isoflavone consumption and development of UC. The association was detected in dietary habits during the previous 1 month, as well as those at 1 year before. Since most UC cases had not experienced their first symptom of UC at 1 year before recruitment, isoflavone consumption at 1 year before seemed to represent an association with pre-illness dietary habits, although the association with dietary habits during the previous 1 month might have included some influence of changes in habits due to disease symptoms.

No previous studies have indicated associations between dietary isoflavone consumption and UC development. Since isoflavones, particularly genistein, show similar binding ability for estrogen receptor β to 17β-estradiol [13], [14] and exert estrogenic activity in some organs [25], [26], it is reasonable to consider that isoflavone consumption might be associated with UC development through similar mechanisms to estrogens. After an estrogen binds to estrogen receptor β in the gut, colonic barrier function is modified [27], [28], which might trigger inappropriate mucosal immune responses. Higher estrogen doses might also cause gastrointestinal ischemia by increasing the tendency for intravascular coagulation [29]. In addition, mucosal inflammation in patients with UC is mediated by Th-2-related cytokines [30], [31], which might offer another plausible biological mechanism for the effect of estrogen. Although the precise pathophysiology of UC remains largely unknown, these estrogen-mediated pathways might play roles in the association between isoflavone consumption and development of UC.

In the present study, gender-stratified analyses indicated that the association with isoflavone consumption was more pronounced in females, but was obscured in males, suggesting that the influence of isoflavone consumption on UC differs across gender. In light of previous studies, one experimental study also demonstrated that perinatal exposure to exogenous estrogens promoted the development of severe intestinal inflammation, particularly among adult female offspring, but not among male offspring [27]. Estrogen-mediated pathways might thus act to promote the development of intestinal inflammation, specifically among females. However, isoflavones exert a wide range of effects as demonstrated in ex vivo model systems and in animals. This already complex picture is further complicated by the fact that pathogenetically relevant in vitro or ex vivo findings do no correlate with the effects of isoflavones in animal models of inflammation [32]. Further epidemiological and experimental studies are thus needed to confirm these findings.

The present study design offered several methodological advantages. First, cases were identified using strict diagnostic criteria and thus the possibility of misclassification of UC was negligible. Second, use of incident cases (newly diagnosed UC patients) minimized the probability of poor recall about pre-illness dietary habits. In addition, to assess the association with pre-illness dietary habits as precisely as possible, we collected information regarding: 1) dietary information on both the previous 1 month and at 1 year before; and 2) the time of first symptom occurrence. This information allowed us to consider the association from several aspects, and sensitivity analyses, in which analyzed UC cases were limited to patients who experienced their first symptom within the preceding 11 months, provided results in which any positive association with isoflavone consumption would be free from reverse causality. Third, the large variation in isoflavone consumption among Japanese individuals allowed the examination of associations between isoflavones and UC, although generalizability to other ethnic groups is uncertain. In fact, daily isoflavone consumption at 1 year before among the present study subjects ranged from 0.01 mg to 138.9 mg (mean daily consumption, 23.6 mg), representing a wider range than seen in other ethnic groups, including US adults (mean daily consumption, 2.6 mg) [33].

However, the following limitations might have influenced the study results. Although response rates among cases and controls were high (84%), non-respondents brought about some variation in matched status, and then provided results with lower power when using conditional logistic regression models limited to matched sets. However, the proportion of non-respondents was similar in both cases and controls, and was considered unrelated to isoflavone consumption. This selection bias might thus have attenuated the association between isoflavone consumption and UC. Second, information bias resulting from imperfect recall of past consumption of soy products might have occurred. However, the hypothesis that soy products are related to UC or inflammatory bowel disease was not recognized by study participants. Thus, all subjects would have received similar recall stimuli about past consumption of soy products. Misclassification due to such information bias, if any, is probably non-differential and would not affect the plausibility of the results. Third, it is also conceivable that other life style characteristics might account for the increasing effects of isoflavone consumption. Although the present results were obtained after adjusting for potential confounders (e.g., body mass index, smoking, and menopausal status) according to the previous study [34], other uncontrolled factors might have affected the validity of our results.

Here, it is important to note that while higher consumption of isoflavones may increase the risk of UC development, the benefits of isoflavones to females may still remain substantial. Several reports have indicated favorable effects of isoflavone intake for decreasing the incidences of breast cancer [35], lung cancer [36] and cerebral or myocardial infarctions [37] and the prevalence of periodontal disease [38]. A major issue is thus the balance between positive and negative health effects of isoflavones. The level of UC risk associated with isoflavone consumption might be rather low. We think that it may be important for clinicians to discuss the possible risks and benefits of isoflavone consumption, especially for females with a family history of UC.

In conclusion, pre-illness isoflavone consumption might be associated with UC development, particularly in females. Prospective cohort studies are needed to confirm these findings.
